# The TLR2/6 ligand PAM2CSK4 is a Th2 polarizing adjuvant in *Leishmania major* and *Brugia malayi* murine vaccine models

**DOI:** 10.1186/s13071-016-1381-0

**Published:** 2016-02-20

**Authors:** Alice Halliday, Joseph D. Turner, Ana Guimarães, Paul A. Bates, Mark J. Taylor

**Affiliations:** Department of Parasitology, Liverpool School of Tropical Medicine, Pembroke Place, Liverpool, L3 5QA UK; Lancaster University, Furness Building, Bailrigg, Lancaster LA1 4YG UK

**Keywords:** Toll-like receptor, Lipopeptide, Adjuvant, Vaccine, *Leishmania*, Lymphatic filariasis

## Abstract

**Background:**

Toll-like receptors (TLRs) play an important role in the innate and adaptive immune responses to pathogens, and are the target of new vaccine adjuvants. TLR2 plays a role in parasite recognition and activation of immune responses during cutaneous leishmaniasis infection, suggesting that TLR2 could be targeted by adjuvants for use in *Leishmania* vaccines. We therefore explored using Pam_2_CSK_4_ (Pam2) and Pam_3_CSK_4_ (Pam3) lipopeptide adjuvants, which activate TLR2/6 and TLR2/1 heterodimers respectively, in vaccine models for parasitic infections.

**Methods:**

The use of lipopeptide adjuvants was explored using two vaccine models. For cutaneous leishmaniasis, the lipopeptide adjuvants Pam2 and Pam3 were compared to that of the Th1-driving double-stranded DNA TLR9 agonist CpG for their ability to improve the efficacy of the autoclaved *Leishmania major* (ALM) vaccine to protect against *L. major* infection. The ability of Pam2 to enhance the efficacy of a soluble *Brugia malayi* microfilariae extract (*Bm*MfE) vaccine to protect against filarial infection was also assessed in a peritoneal infection model of *B. malayi* filariasis. Parasite antigen-specific cellular and humoral immune responses were assessed post-challenge.

**Results:**

The use of lipopeptides in ALM-containing vaccines did not provide any protection upon infection with *L. major*, and Pam2 exacerbated the disease severity in vaccinated mice post-challenge. Pam2, and to a lesser extent Pam3, were able to elevate antigen-specific immune responses post-challenge in this model, but these responses displayed a skewed Th2 phenotype as characterised by elevated levels of IgG1. In the *B. malayi* vaccine model, the use of Pam2 as an adjuvant with *Bm*MfE induced significant protective immunity to the same level as inclusion of an Alum adjuvant. Here, both Pam2 and Alum were found to enhance antigen-specific antibody production post-challenge, and Pam2 significantly elevated levels of antigen-specific IL-4, IL-5 and IL-13 produced by splenocytes.

**Conclusions:**

These data indicate that TLR2/6-targeting ligands could be considered as adjuvants for vaccines that require robust Th2 and/or antibody-dependent immunity.

## Background

Toll-like receptor (TLR)s are innate immune receptors which recognise distinct molecular patterns (pathogen-associated molecular patterns; PAMPs) of microbial organisms. Due to their ability to promote strong innate and adaptive immune responses, TLR ligands are a promising new class of adjuvants [1]. Many TLR ligand formulations have been included in experimental vaccines in human clinical trials, implicating them as safe and efficacious adjuvants, but so far only two, which both contain the TLR4 ligand 3-O-desacyl-4’-monophosphoryl lipid A (MPL), have been licensed for use in human vaccines: the Adjuvant System 04 (ASO4; GlaxoSmithKline) is an oil-in water adjuvant and is licensed for use in the human papilloma virus (HPV) vaccine, and RC-529 which contains Alum and is licenced for use in the hepatitis B vaccine [1–3]. Clearance of parasitic infections requires different types of adaptive immune response depending on the type of causative pathogen, with intracellular protozoa (such as *Plasmodium* and *Leishmania spp*) requiring a Th1-type immune response, while extracellular parasitic helminths (such as filarial nematodes) require a Th2 response. Thus, the use of parasitic models of infection in preclinical vaccine studies can allow us to explore the ability of different vaccine and adjuvant formulations to drive immune responses post challenge. Some TLR ligands, such as various TLR4 ligands and the TLR9 ligand CpG, have shown good efficacy in preclinical vaccine studies of malaria and leishmaniasis [4–8].

TLR2 has been implicated in the recognition of *Leishmania* parasites [9, 10], and in playing a role during infection in vivo [11]. A widely used vaccine model for cutaneous leishmaniasis is heat-killed autoclaved *L. major* (ALM) given in two doses (prime and boost) prior to challenge with *L. major* promastigotes [7, 12–14]. In mice, the ALM vaccine provides good efficacy to challenge infections with *Leishmania* parasites [7, 15]. CpG, an agonist of TLR9, elicits a strong Th1 response to a killed whole-cell *L. major* vaccine in susceptible BALB/c mice, and provides protection in 40 % of animals receiving the vaccine [12]. Whilst the ability of whole-cell *Leishmania* vaccines to provide long term protective immunity has been disputed, Okwor et al. [15] demonstrated that repeated inoculation with ALM could result in the expansion of sufficient Th1 memory T cells specific for *L. major* and this strategy was as effective as live parasites at providing protection to challenge up to 13 weeks after the final dose was given. This demonstrates that the use of first generation killed parasite vaccines can provide protection to *L. major* when delivered in the appropriate manner, and stimulates the search for the most appropriate adjuvants to increase the efficacy of these vaccines.

TLR2 and its co-receptor TLR6 have also been found to play a role in disease pathogenesis during infection with the filarial parasite, *Brugia malayi*, which causes lymphatic filariasis. In this setting, TLR2 is activated by a lipopeptide from the bacterial endosymbiont of *B. malayi*, *Wolbachia* [16, 17]. Mouse models to assess the efficacy of experimental vaccines to protect against lymphatic filariasis have used various forms of antigen, including recombinant proteins, DNA vaccines, and attenuated larvae, of which the latter has had the greatest success [18, 19]. Given that using attenuated parasite larvae is not feasible for a licensed vaccine, finding an alternative approach using protein and adjuvant combinations would be advantageous. As with resistance to primary infections, both T and B cell responses appear to be crucial for enhanced clearance to filarial infections in vaccinated mice, as well as the production of IL-5 and subsequent recruitment of eosinophils [20–22].

Lipopeptides are agonists for TLR2 and their ability to stimulate both cellular and humoural immune responses has been widely reported [23–25], but their use as adjuvants in vaccines for parasitic infections remains relatively unexplored. In this study, we aimed to explore the use of lipopeptides as potential new adjuvants for use in vaccine models against two different parasitic infections: *Leishmania major* which requires Th1 immunity, and *Brugia malayi*, which requires Th2 immunity [26].

## Methods

### Mice and parasites

All procedures involving the use of laboratory animals were performed at the Biomedical Services Unit (BSU), University of Liverpool, were approved by the Ethics and Animal Care Committees of the University of Liverpool and Liverpool School of Tropical Medicine (LSTM), and were carried out according to the Animals (Scientific Procedures) Act (UK Home Office).

Female C57BL/6 mice were purchased from Charles River (UK) for use in *Leishmania* infection/ vaccine experiments, while male BALB/c mice were purchased from Harlan (UK) for *B. malayi* infection/ vaccine experiments; all were 8–10 weeks at the start of each experiment. Animals were randomly allocated in to groups for vaccination and challenge experiments. While downstream analysis on the outcomes of animal experiments was not performed blind to experimental groups, individual samples from different groups were mixed during measurement and analysis, to ensure no bias was introduced.

*L. major* FV1 (MHOM/IL/80/Friedlin) promastigote parasites were cultured in complete M199 medium (Invitrogen, containing 10–20 % heat-inactivated foetal calf serum (PAA), BME vitamins (Sigma), and 25 μg/ml gentamicin sulphate (Sigma)) and were sub-passaged no more than twice after initial culture of lesion-derived amastigotes. For the infectious challenge, parasites were enriched for metacyclics as described elsewhere [27].

The *B. malayi* life cycle was maintained at LSTM as described [18]. Jirds infected in the peritoneum with adult *B. malayi* parasites were originally purchased from TRS laboratories, USA. Microfilariae (Mf) produced by *B. malayi* adults were obtained by a peritoneal tapping method, as described by Griffiths [28].

### *Leishmania* whole cell vaccines, vaccination and challenge infection

Autoclaved *L. major* (ALM) antigen was made using a method described first by Bahar et al. [29]. Briefly, *L. major* promastigote cultures were grown to log-phase (day 5) in complete M199 and diluted 1:5 in complete Grace’s medium for a further 4 days. The promastigote parasites (approximately 10^9^) were then washed (X3) in sterile phosphate buffered saline (PBS) and resuspended in 2 ml in a glass container and autoclaved at 151 °C for 15 minutes. Protein concentration was measured using the BCA assay (Pierce/ThermoScientific) and aliquots were stored at –80 °C. The unmethylated CpG Oligodeoxynucleotide (ODN) 1826 adjuvant of the sequence 5’- TCCATGACGTTCCTGACGTT -3’ (CpG) was a kind gift from Lyn Jones and Matthew Selby at Coley (Pfizer). Lipopeptide adjuvants S-[2,3-bis(palmitoyloxy)-(2*RS*)-propyl]-(*R*)-cysteine (Pam2) and N-Palmitoyl-S-[2,3-bis(palmitoyloxy)-(2*RS*)-propyl]-(*R*)-cysteine (Pam3) were purchased from EMC Microcollections. CpG, Pam2 and Pam3 were dissolved in nuclease-free water and stored as 1–10 mg/ml stocks at –80 °C.

Mice were immunised with 20 μl of PBS alone; 50 μg ALM; 50 μg ALM + 50 μg CpG; 50 μg ALM + 10 μg Pam2; 50 μg ALM + 10 μg Pam3. The first dose was given s.c to the upper side of the left hind foot (LHF), and the second dose was given s.c to the shaven rump two weeks later. Four weeks after the second vaccine dose, mice were challenged by s.c. injection of 10^5^ metacyclic-enriched *L. major* FV1 parasites, in 20 μl Hank’s Balanced Salt solution (HBSS, Sigma), to the upper side of the right hind foot (RHF).

Measurement of lesion size on the infected foot was achieved by measuring the thickness of the two hind feet using a dial calliper and subtracting the thickness of the uninfected foot from that of the infected foot (mm).

### *B. malayi* vaccines, vaccinations and challenge infection

*B. malayi* Mf parasite extract (*Bm*MfE) was prepared as previously described [30] with some adjustments. Mf were separated from the peritoneal exudate using a PD-10 column (GE Life Sciences) and resuspended in sterile PBS at 2 × 10^6^/ml prior to sonication and centrifugation [30]. Imject® Alum Adjuvant (Alum, Thermo Scientific) was added dropwise to *Bm*MfE to a ratio of 1:1 and mixed for 30 min at 4 °C. Mice were immunised with 100 μl s.c at the nape of the neck with either: PBS; 50 μg *Bm*MfE; 50 μg *Bm*MfE + 10 μg Pam2; 50 μg *Bm*MfE + Alum. Two weeks later, mice were challenged with 50 *B. malayi* L3 by i.p injection. Parasites were recovered six or nine days post challenge.

### Immune responses

The levels of antigen specific IgG1, IgG2c (C57BL/6 mice) and IgG2a (BALB/c mice) in plasma samples from mice were measured using a sandwich antibody ELISA (Bethyl Laboratories) with *L. major* freeze thaw antigen (FTAg; *Leishmania* experiments) or *Bm*MfE; (*B. malayi* experiments) as the capture antigen at a concentration of 10 μg/ml.

To assess recall responses, splenocytes were used at a concentration of 8 × 10^5^ cells/ well in complete medium for 72 h in the presence of parasite antigens (10 μg/ml FTAg or 20 μg *B. malayi* L3 extract; *Bm*L3E), or medium alone (negative control), in a total volume of 200 μl/well. Culture supernatants were stored at –20 °C until analysis for cytokine (interferon(IFN)γ, interleukin (IL)-4, IL-5 and IL-13) levels using a sandwich cytokine ELISA (R&D Systems).

### Statistical analysis

Where datasets were normally distributed (as determined using the Shapiro-Wilk test), variance between groups was determined using a one-way ANOVA and Dunnet’s post-hoc test to compare test groups to control. Where datasets were found to be non-Gaussian, variation across groups was analysed using the Kruskall-Wallis test with the Dunn’s post hoc-test to compare pairs of groups. Statistical analysis was conducted using SPSS and Graphpad Prism Software with a significance level of *p* < 0.05.

## Results

### Lipopeptide adjuvant Pam2 neutralises protection and exacerbates disease in *L. major* vaccines

**Fig. 1:**
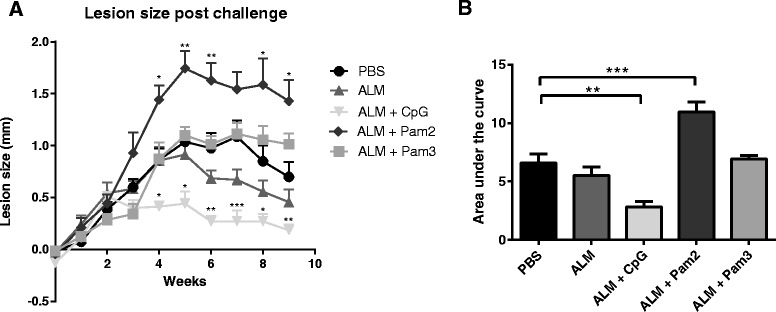
Development of *L. major* disease in mice vaccinated with ALM and adjuvants. Groups of 7-8 mice were vaccinated twice with one of the following formulations: PBS; ALM; ALM + CpG; ALM + Pam2; ALM + Pam3 and were subsequently challenged four weeks after the second dose with *L. major* promastigotes in the right hind foot (RHF). **a** Lesion development was monitored by measuring the difference in thickness of the infected and uninfected feet (RHF-LHF) in mm; mean values + SEM are shown (* *p* <0.05; *p* < 0.005 **; *p* < 0.0001). **b** The overall disease severity is summarised by calculating the area under the curve (AUC) from weekly lesion size data sets (mean + SEM is shown). A single experiment was performed, adequately powered to detect a difference of < 40 % between lesion sizes in different groups. For both datasets, normality was observed and thus variance between groups was determined using a one way ANOVA, with Dunnet’s post hoc test to determine differences between test groups and the PBS control (* *p* <0.05; *p* < 0.005 **; *p* < 0.0001)

Mice vaccinated with ALM alone showed a partial but non-significant reduction in lesion size, with no significant difference in the AUC values between ALM-vaccinated and the PBS-vaccinated control group (Fig. 1A&B). When the gold standard CpG adjuvant was included in the ALM vaccine, mice developed significantly reduced lesions compared to either PBS- or ALM-vaccinated mice, with reduced lesion sizes from 4-9 weeks post infection and significantly reduced AUCs. The addition of Pam3 appeared to neutralise the partial reduction of ALM alone and showed a similar profile to PBS control group. Notably, when Pam2 was used with ALM, the lesion sizes were significantly increased when compared to sham vaccinated (PBS) mice from 4 weeks p.i., and the AUC values were also significantly increased (Fig. 1A&B). Thus, when compared to the gold standard TLR agonist adjuvant CpG, lipopeptide adjuvants have an opposing effect on lesion development post-challenge when used in a vaccine for cutaneous leishmaniasis.

### Pam2 drives a predominantly Th2 type immune response characterised by elevated IgG1 levels following ALM vaccination

In order to determine whether there were differences in the magnitude or polarization of adaptive immune responses post-challenge, we measured the ratio of antigen-specific IgG1:IgG2c antibody levels as a marker of the Th1/Th2 polarisation [31]. Antigen-specific IgG1 responses were significantly elevated in the ALM + Pam2 vaccinated mice (and not ALM vaccinated mice), when compared to PBS (sham) vaccinated controls, suggesting that the Pam2 lipopeptide adjuvant results in elevated Th2 antibody responses.

**Fig. 2:**
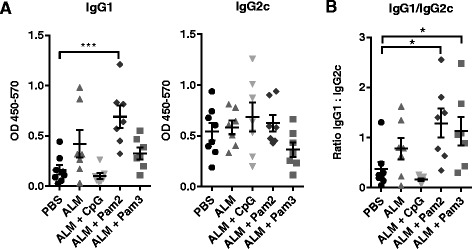
Antigen specific IgG antibody responses to FTAg in vaccinated mice infected with *L. major* for 9 weeks. Groups of 7-8 mice were vaccinated twice with one of the following formulations: PBS; ALM; ALM + CpG; ALM + Pam2; ALM + Pam3 and challenged four weeks later with *L. major*. Plasma samples were collected after 9 weeks of infection with *L. major*. **a** Levels of IgG1 and IgG2c antibodies specific for *L. major* FTAg were determined by ELISA, results are displayed as Absorbance (Abs) at 450-570 nm; bars represent the mean + SEM values for the average levels of antibody (from duplicate samples for each individual) in each group. **b** The ratio of IgG1:IgG2c was estimated using the mean absorbance values for each individual (from supplicate values); points represent the ratio of mean antibody levels (IgG1:IgG2c) for each individual. A single experiment was performed, adequately powered to detect a difference of < 40 % between lesion sizes in different groups. Variation between groups was determined by conducting a one-way ANOVA test with Dunnet’s post hoc test to compare test groups with controls (*p* < 0.05 *; *p* < 0.005 **; *p* < 0.0001)

When Pam2 or Pam3 was included in an ALM vaccine, there was a significant shift towards a Th2 response to *L. major* antigen when compared to PBS- vaccinated mice (Fig. 2B), as demonstrated by elevated IgG1:IgG2c levels in the plasma. In contrast, those vaccinated with ALM + CpG had the lowest IgG1:IgG2c ratio compared to all other groups (*p* < 0.005). Thus, both the lipopeptide adjuvants skewed the immune response towards a Th2 type when compared to CpG adjuvant or when mice were unexposed to antigen prior to infection, based on parasite-specific antibody profiling. Levels of Th1 and Th2 cytokines were measured after in vitro stimulation of splenocytes with *L .major* antigen FTAg, but the results failed to demonstrate clear differences in adaptive immune responses between groups (data not shown). This is likely due to the late time point during infection (week 9) at which the splenocytes were recovered, as at this point all groups have a predominant Th1 response, have begun to control the infection and reduce lesion sizes.

### Pam2 reduces parasite burden and drives a Th2 response in a vaccine model of filariasis

**Fig. 3:**
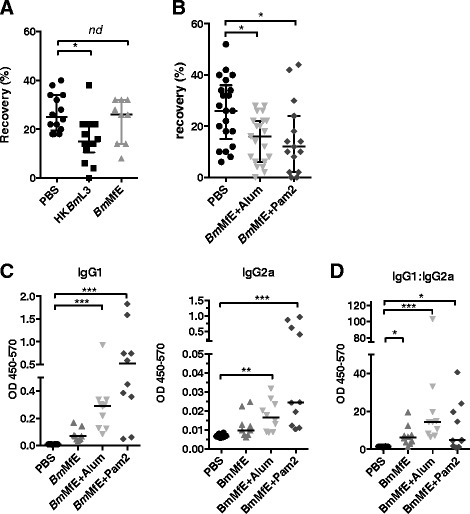
The use of Pam2 adjuvant in a vaccine model for filariasis. Groups of 5 BALB/c mice were vaccinated with either a PBS control, HK*Bm*L3 twice, 14 days apart or formulations containing *Bm*MfE with or without the test adjuvants Pam2 or Alum once, s.c. to the nape of the neck. Fourteen days after final vaccination, mice were challenged by infection of 50 *B. malayi* L3 larvae by intraperitoneal injection. **a** & **b** Parasite recoveries 6 days after infection are presented, data is pooled from 2-3 individual experiments; median +/- interquartile is shown. **c** At the time of challenge recovery, blood and plasma were collected and plasma samples were used to measure the amount of circulating IgG antibody isotypes specific for the vaccine antigen *Bm*MfE; levels of relative IgG1 and IgG2a are shown. **d** The ratio of IgG1:IgG2a levels is also shown. Data presented is pooled from 2 experiments; median averages are shown. Variation between groups was determined by conducting a Kruskall-wallis test with Dunn’s post hoc test to compare test groups with PBS controls (*p* < 0.05 *; *p* < 0.005 **; *p* < 0.001***)

Because of the exacerbation of disease and Th2-skewed immune profile in the context of *Leishmania* vaccination, we subsequently tested the use of Pam2 as an adjuvant for vaccines that require Th2 responses for protective immunity, by utilising a vaccine model of the human lymphatic filaria, *B. malayi*. Four weeks prior to challenge infection mice were vaccinated with either PBS, a gold-standard whole heat-killed *Brugia* L3 larval preparation (HK*Bm*L3), a filarial antigen extract (*Bm*MfE), *Bm*MfE + Alum, or *Bm*MfE + Pam2. For HK*Bm*L3, mice received a boost two weeks prior to challenge. Mice vaccinated with the filarial extract *Bm*MfE alone showed non-significant reduction in the average parasite recovery in contrast with the gold-standard HK*Bm*L3 vaccine (Fig. 3A). However, when mice were vaccinated with *Bm*MfE + Alum, a significant mean 43 % reduction in parasite recovery compared to challenge controls was observed (Fig. 3B). When the Pam2 adjuvant was used, a similar, mean (41 %) significant reduction in parasite recovery was observed. Thus, Pam2 adjuvancy was able to reduce parasite burdens to levels comparable to the gold standard Th2-driving Alum.

The greatest IgG1 responses were recorded in individuals vaccinated with *Bm*MfE + Pam2 (0.83 +/- 0.26), followed by *Bm*MfE + Alum (0.413 +/- 0.129) and *Bm*MfE (0.071 +/- 0.019), and both groups vaccinated with adjuvants produced significantly higher levels of IgG1 compared to those vaccinated with PBS alone (Fig. 3B). Thus Pam2 is able to induce a more pronounced increase in the antigen specific IgG1 response when compared to Alum.

**Fig. 4:**
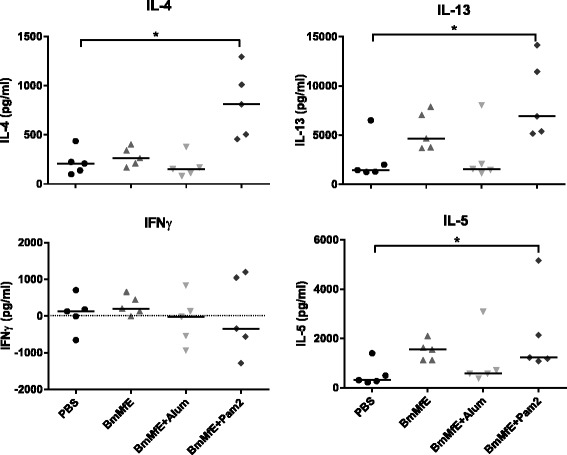
L3 specific splenocyte cytokine responses of vaccinated mice and controls in the *B. malayi* vaccine model. Splenocytes were recovered from vaccinated mice challenged with *B. malayi* L3 larvae, 6 days after challenge. Levels of cytokine (IL-4, IL-13, IFNγ and IL-5) produced by unstimulated cells were subtracted from those produced in the presence of *Bm*L3E. Those sham vaccinated with PBS are shown (black circles), as well as those vaccinated with *Bm*MfE, *Bm*MfE + Pam2, and *Bm*MfE + Alum (grey triangles, light grey inverted triangles, and dark grey diamonds respectively). Individual points represent the mean average levels of cytokine calculated from triplicate splenocyte cultures per mouse, and horizontal bars represent median averages for each group. Data presented is from one individual experiment but is representative of 2. Variation between groups was determined by conducting a Kruskall-wallis test with Dunn’s post hoc test to compare test groups with PBS controls (*p* < 0.05 *)

The levels of antigen specific IgG2a antibody were also elevated in the *Bm*MfE + Pam2 group and *Bm*MfE + Alum group compared to the sham vaccinated mice, indicating that Pam2 is able to drive a mixed Th1/Th2 response (Fig. 3B). This was further reflected in the ratios of IgG1:IgG2a levels found in the plasma of the vaccinated mice, with the highest elevated levels recorded in the group which received the Alum adjuvant, but with all of the vaccine groups showing elevated IgG1:IgG2a levels compared to PBS controls. Thus, Pam2 is a strong driver of both IgG1 and IgG2a responses, and is comparable to Alum in its ability to drive strong antibody responses. When splenocytes were isolated from challenged mice and re-stimulated with infectious-stage parasite antigen (*B. malayi* L3 extract; *Bm*L3E) cytokine production indicated a significant elevation of Th2 cytokines; IL-4, IL-13 and IL-5 (but not the Th1 cytokine IFNγ) in mice vaccinated with *Bm*MfE + Pam2. In contrast, vaccination with Alum had no effect on cytokine recall responses at the time-point assayed (Fig. 4).

## Discussion

Lipopeptides are TLR2-activating molecules that have potential use as new adjuvants for vaccines [24, 32, 33]. They can induce both Th1 and Th2 immunity and are particularly effective at promoting antibody and cytotoxic T-lymphocyte (CTL) effector and memory responses through enhanced priming of dendritic cells [34]. Here we explored their use in two different models of parasitic vaccination, in which protective immunity is polarised towards either Th1 (*L. major*) or Th2 (*B. malayi*) immunity.

The rationale for evaluating lipopeptides as adjuvants for *Leishmania* vaccines is based on our previous work, which demonstrates a protective role for TLR2 in infections with both *L. major* and *L. mexicana* (Halliday et al. manuscript in preparation). We hypothesised that TLR2-activating adjuvants would enhance the efficacy of whole cell *Leishmania* vaccines*.* However, we observed the opposite effect with lipopeptide adjuvants, which neutralised vaccine efficacy and, in the case of Pam2, exacerbated the severity of challenge infection. Whilst this is in contrast to a previous study that showed a lipopeptide-containing recombinant vaccine gave enhanced protection against *L. major* challenge and drove a strong Th1 immune response [35], trace endotoxin contaminants and other PAMPs derived from the expression system may explain difference in outcome. In our study, pure, synthetic lipopeptides resulted in the promotion of Th2 immune responses over the Th1 response, in contrast to the Th1 driving CpG adjuvant, which provided increased protection to *L. major*. This was manifest in the ratio of IgG1:IgG2c levels in the ALM + Pam2 and to a lesser extent ALM + Pam3 vaccinated mice, which were skewed in favour of IgG1. Several studies have linked *Leishmania*-specific IgG [36, 37], and in particular IgG1 antibody isotypes [38] to susceptibility to infection with *L. major* or other *Leishmania spp*. During infection, amastigotes are able to infect new macrophages via IgG antibody receptors (FcγRs), resulting in production of IL-10 at the site of infection and allowing further parasite replication [39]. The elevated IgG1 levels in the Pam2 vaccinated mice therefore may have a direct role for the elevated disease severity observed in these mice. Alternatively, lipopeptide-driven expansion of Foxp3^+^ CD25^+^ CD4^+^ regulatory T cells may also compromise protective Th1 immunity [40]. The finding that Pam2 has a higher potency in the *L. major* model when compared to Pam3 (in terms of enhancing disease severity and driving antigen-specific antibody responses), is consistent with the findings of others [41], and may relate to the higher solubility characteristics of Pam2.

Conversely to the deleterious effects in the *L. major* vaccine model, when Pam2 was included in the *B. malayi* vaccine, it promoted protective immunity with similar efficacy to Alum adjuvants. In the *B. malayi* vaccine, elevated IgG1 levels were also observed in challenged mice when Pam2 was included in the vaccine. In addition, an elevated production of IL-4, IL-13 and IL-5 was observed after the ex vivo stimulation of splenocytes, again showing an elevated Th2 response with Pam2. An elevated level of antigen-specific IgG2a was also detected in mice vaccinated with the *B. malayi* + Pam2 vaccine, which was not observed in the *L. major* model (for IgG2c). This may reflect a differential response to the Pam2 adjuvant between the strains of mice used (C57BL/6 vs BALB/c), to the difference in antigen and/or challenge pathogen used in the model, or indeed to the chronicity of challenge infection (9 weeks or 6-9 days respectively). Importantly, whilst both Alum and Pam2 elevated both antigen specific IgG1 and IgG2a levels in the *B.malayi* model, the ratio of IgG1:IgG2a in *B. malayi* + Pam2 vaccinated mice was lower compared to Alum, indicating that Alum is a more selective driver of Th2 rather than Th1 responses (reported before in mice [42]). Alum adjuvants are able to stimulate enhanced innate immune responses at the site of exposure, in a mechanism independent of TLR signalling (via MyD88 and/or Trif) [43]. Thus, while Alum and Pam2 both act to enhance innate and adaptive immune responses, the mechanism of action by which they do this is strikingly different, as Pam2 drives immune responses via engaging with TLR2/6, and via MyD88 signalling [44]. Mechanisms which have been attributed to Alum’s ability to potentiate immune responses include activation of the NOD-like receptor family, pyrin-domain-containing 3 (NLRP3) inflammasome [45], and induction of cell death followed by subsequent release of endogenous danger signals [46]. However, the importance of each of these in the ability of Alum to drive adaptive immune responses is still a subject of debate, as some groups have yet to find evidence of the involvement of the NLRP3 inflammasome upon exposure to Alum [46–48].

Humoral responses have important roles in parasite clearance in lymphatic filariasis [49, 50]. B cell deficient mice were unable to reduce parasite burden after pre-exposure to irradiated L3 vaccine [50]. A recent study by Sharmila et al. explored the use of a recombinant lipidated antigen of filarial parasites, abundant larval transcript (ALT), in a rodent model of *B. malayi* infection [51]. This study corroborates our findings as they also demonstrate that the addition of a free diacylated lipopeptide adjuvant can enhance protection, and drive increased antibody and cytokine responses when included in a vaccine with native protein antigen [51]. It is interesting that studies exploring the use of vaccines containing recombinant antigens engineered to harbour intrinsically-linked lipid adjuvants report that such formulations are able to drive strong immune response of a Th1-type, in models of both *L. major* and *B. malayi* [35, 51]. Thus, the influence of a lipopeptide adjuvant on the resultant immune responses may differ dramatically depending on the way in which it is presented; with free lipopeptide adjuvants favouring an antibody and mixed Th1/Th2 response, whilst intrinsically linked antigen and lipopeptide formulations favour a Th1 response. Further, free lipopeptides formulated so that they can electrostatically attach to antigens appear to favour enhanced cellular immunity [34].

Previous studies exploring the use of lipopeptide adjuvants have reported various types of immune responses, ranging from elevated IL-10 and/or T regulatory cell responses [40, 52, 53], to enhanced Th1 responses [54–56]. Pandey et al. recently demonstrated that while Pam3 and peptidoglycan (PGN) can enhance infection of *L. major* in macrophages in vitro, a diacylated lipopeptide BPPcysMPEG can reduce infection [55]. Furthermore, BPPcysMPEG was able to reduce *L. major* infection in both prophylactic and therapeutic settings in a BALB/c model [55], which is in contrast to the enhanced diseased caused by Pam2 in our experiments. It is evident that the conflicting findings in the literature on the immune response elicited by lipopeptide adjuvants, suggests that the subsequent immune response is not an intrinsic property of the lipopeptides but is influenced by the lipopeptide-vaccine combination. This contrasts with other TLR ligand adjuvants, which consistently drive strong Th1 immunity [1]. Further research to define under which circumstances lipopeptide adjuvants promote polarised or mixed Th1/Th2 immunity are required to support rational vaccine design [57, 58].

## Conclusions

In summary, this study indicates that the use of Pam2 as an adjuvant is able to drive improved efficacy in a helminth vaccine model where Th2 immunity is required for protective immunity, but is detrimental in vaccines requiring Th1 immunity.

## Abbreviations

ALM: autoclaved *Leishmania major*
ANOVA: Analysis of variation
AUC: area under the curve
*Bm*L3E: *B. malayi* L3 extract
*Bm*MfE: *B. malayi* Mf extract
CTL: cytotoxic T lymphocyte
FcγRs: IgG antibody receptors
IFNγ: interferon gamma
IgG: gamma immunoglobulin
IL: interleukin
L3: third-stage larvae
Mf: microfilariae
Pam2: Pam_2_CSK_4_
Pam3: Pam_3_CSK_4_
PAMPs: pathogen-associated molecular patterns
PBS: phosphate-buffered saline
Th: T-helper cell
TLRs: Toll-like receptors
